# Functional macronutritional generalism in a large omnivore, the brown bear

**DOI:** 10.1002/ece3.3867

**Published:** 2018-01-29

**Authors:** Sean C. P. Coogan, David Raubenheimer, Gordon B. Stenhouse, Nicholas C. Coops, Scott E. Nielsen

**Affiliations:** ^1^ Department of Renewable Resources University of Alberta Edmonton AB Canada; ^2^ Faculty of Life and Environmental Sciences, and the Charles Perkins Centre University of Sydney Sydney NSW Australia; ^3^ Grizzly Bear Program, fRI Research Hinton AB Canada; ^4^ Department of Forest Resource Management University of British Columbia Vancouver BC Canada

**Keywords:** generalist, grizzly bear, macronutrients, niche breadth, omnivore, *Ursus arctos*

## Abstract

We combine a recently developed framework for describing dietary generalism with compositional data analysis to examine patterns of omnivory in a large widely distributed mammal. Using the brown bear (*Ursus arctos*) as a model species, we collected and analyzed data from the literature to estimate the proportions of macronutrients (protein, carbohydrate, and lipid) in the diets of bear populations. Across their range, bears consumed a diversity of foods that resulted in annual population diets that varied in macronutrient proportions, suggesting a wide fundamental macronutrient niche. The variance matrix of pairwise macronutrient log‐ratios indicated that the most variable macronutrient among diets was carbohydrate, while protein and lipid were more proportional or codependent (i.e., relatively more constant log‐ratios). Populations that consumed anthropogenic foods, such agricultural crops and supplementary feed (e.g., corn), had a higher geometric mean proportion of carbohydrate, and lower proportion of protein, in annual diets. Seasonally, mean diets were lower in protein and higher in carbohydrate, during autumn compared to spring. Populations with anthropogenic subsidies, however, had higher mean proportions of carbohydrate and lower protein, across seasons compared to populations with natural diets. Proportions of macronutrients similar to those selected in experiments by captive brown bears, and which optimized primarily fat mass gain, were observed among hyperphagic prehibernation autumn diets. However, the majority of these were from populations consuming anthropogenic foods, while diets of natural populations were more variable and typically higher in protein. Some anthropogenic diets were close to the proportions selected by captive bears during summer. Our results suggest that omnivory in brown bears is a functional adaptation enabling them to occupy a diverse range of habitats and tolerate variation in the nutritional composition and availability of food resources. Furthermore, we show that populations consuming human‐sourced foods have different dietary macronutrient proportions relative to populations with natural diets.

## 1 INTRODUCTION

Diet is a primary factor used to characterize the ecological niches of species and populations, including classification along the generalist–specialist spectrum (Elton, 2001; Hutchinson, 1957). However, dietary generalism usually is coarsely characterized, with the role of nutrients in defining a species’ niche overlooked (Machovsky‐Capuska, Senior, Simpson, & Raubenheimer, 2016). This is problematic because nutrition plays a dominant role in determining which foods species consume, and thus which environments they inhabit (Raubenheimer, Simpson, & Tait, 2012). The field of nutritional ecology has demonstrated that, in particular, the macronutrients (protein, carbohydrate, and lipid) in the foods and diets of animals strongly influence their foraging behavior (Coogan et al., 2017; Rothman, Plumptre, Dierenfeld, & Pell, 2007) and ultimately fitness, including reproduction and longevity (Jensen et al., 2012; Solon‐Biet et al., 2014). Neglecting nutrition in niche theory might therefore limit our understanding of the ecological factors that determine the abundance and distribution of species.

Recently, a multidimensional framework was developed for integrating nutrition and ecological niche theory (Machovsky‐Capuska, Senior, et al., 2016). This approach characterizes the dietary niche of species across four functional levels: (1) the diversity of physical and ecological characteristics of foods a species can utilize (“food exploitation niche”); (2) the range of food macronutrient compositions a species can consume as part of its diet (“food composition niche”); (3) the range of dietary macronutrient compositions that a species is physiologically capable of persisting on (“fundamental macronutrient niche”); and (4) the range that it actually persists on, given ecological constraints such as food availability and competition (“realized macronutrient niche”).

The purpose of this study was to investigate patterns of dietary generalism in a large widely distributed mammalian omnivore, through the lens of macronutritional niche theory. To that end, we selected the brown bear (*Ursus arctos*) as an exemplar model species. The brown bear has an ecologically and geographically wide (i.e., circumpolar) distribution (Pasitschniak‐Arts, 1993). Across its range, the brown bear has a polyphagous diet consisting of a wide range of foods that vary in physical, ecological, and nutritional properties, depending upon both seasonal and local availability (Bojarska & Selva, 2012; Coogan, Raubenheimer, Stenhouse, & Nielsen, 2014). As apex predators, brown bears consume a range of animal prey, such as small and large mammals, insects, fish, and birds (e.g., Ciucci, Tosoni, Di Domenico, Quattrociocchi, & Boitani, 2014; Rigg & Gorman, 2005). Brown bears also consume a variety of graminoids and forbs, consume both soft mast (i.e., fruit) and hard mast (i.e., nuts), and possess the ability to dig for and consume belowground vegetation (e.g., roots and corms, Hamer & Herrero, 1987). Brown bears are also known to obtain a variety of both plant‐ and animal‐based foods from anthropogenic sources, including grain, livestock, and human food waste (Gunther et al., 2004; Murray, Fassina, Hopkins, Whittington, & St Clair, 2017). In the context of the multidimensional nutritional niche, the brown bear can thus be characterized as a generalist in both food exploitation (level 1 above) and food composition (level 2 above).

What are not known, however, are the fundamental and realized macronutrient niches (levels 3 and 4 above) of brown bear, which are important for understanding the relationships between their nutritional environments, adaptation, population persistence, and functional omnivory more generally. The fundamental macronutrient niche concept is also germane to understanding and implementing applied ecology, such as whether a population (or individual) is likely to persist in the face of substantial perturbations to its nutritional environment, due to factors such as climate change, translocation, and dispersal.

A macronutrient self‐selection study of captive brown bears found that they preferred a ratio of 17% protein to 83% nonprotein (carbohydrate + lipid) metabolizable energy (Erlenbach, Rode, Raubenheimer, & Robbins, 2014). The study provided strong evidence that this ratio is functionally significant, because compared with other dietary compositions it maximized mass gain (primarily fat mass) per unit energy intake, which is an important outcome for a hibernating mammal. Bears in that study preferred high‐lipid intake, but when confined to low‐fat diets would maintain the target ratio of protein to nonprotein energy by consuming carbohydrate.

Brown bears in the wild, however, may be precluded from foraging to meet such nutritional preferences, because foods high in lipid or carbohydrate necessary to maintain a balanced intake are generally most available during late summer and autumn (Coogan et al., 2014). That foods available to achieve the optimal macronutrient ratio for primarily fat mass gain co‐occur with the prehibernation hyperphagic period, is suggestive of the functional significance and selective pressures shaping their behavioral dietary preferences; the nutritional and energetic demands necessary for hibernation require the acquisition of sufficient food resources (Rigano et al., 2017), with higher demands for females birthing cubs (López‐Alfaro, Robbins, Zedrosser, & Nielsen, 2013). It is unclear, however, the extent to which bears regulate their diets in the wild.

Here, we infer the minimal fundamental macronutrient niche of brown bear using population diet estimates as indicators of realized macronutrient niches. To that end, we collected and synthesized data from the literature to estimate the proportions of macronutrients in the diets of brown bear populations, following the approach recently applied to a small carnivore (*Martes martes*; Remonti, Balestrieri, Raubenheimer, & Saino, 2016) and invasive omnivore (*Sus scrofa*; Senior, Grueber, Machovsky‐Capuska, Simpson, & Raubenheimer, 2016). We then applied a compositional statistical paradigm (Aitchison, 1982) to our data analysis of macronutrient proportions. We hypothesized that the brown bear's broad diet could be associated with the following functional adaptations of omnivory:

1. The *Nutrient Balancing Hypothesis* predicts that a wide diet serves to increase the range of food options that can be combined to achieve a balanced macronutrient intake. This hypothesis predicts that the macronutrient composition of bear diets from different ecological and geographic populations is similar despite consuming different foods (i.e., a narrow fundamental macronutrient niche). This type of nutrient balancing has been observed in primates (Raubenheimer, Machovsky‐Capuska, Chapman, & Rothman, 2015; Rothman et al., 2007), badger (*Meles meles*; Remonti, Balestrieri, & Prigioni, 2011), and pine marten (*Martes martes*; Remonti et al., 2016).
2. The *Nutritional Generalism Hypothesis* postulates that a broad diet combined with the ability to tolerate a wide range of dietary macronutrient intakes enables a species to occupy a diverse range of habitats. This hypothesis predicts variation in brown bear diet compositions among populations and a wide fundamental macronutrient niche. This type of nutrient balancing has been observed in gannets (*Morus* spp.; Tait, Raubenheimer, Stockin, Merriman, & Machovsky‐Capuska, 2014) and wild boar (Senior et al., 2016).
3. The *Seasonal Variation Hypothesis* predicts that the proportion of macronutrients in the diets of brown bear will vary seasonally (Coogan et al., 2014). It is well known that the protein content of bear diets declines over the active season in several ecosystems (López‐Alfaro, Coogan, Robbins, Fortin, & Nielsen, 2015). However, the multivariate relationship between seasonal macronutrient proportions has been less well established (but see Coogan et al., 2014 and Costello et al., 2016). This hypothesis is nonmutually exclusive with either of the above hypotheses.
4. The *Prehibernation Optimal Diet Hypothesis* predicts that the macronutrient composition of brown bear diets will be closer to the self‐selected optimal ratio for mass gain of captive bears during the prehibernation hyperphagic season, because selective pressure during this period will be the highest (i.e., behavioral and physiological adaptation).

Furthermore, in addition to hypotheses specific to omnivory, we test the hypothesis that bear diets documenting anthropogenic food “subsidies” (e.g., livestock, agricultural crops, and supplemental feeds) are higher in nonprotein macronutrients than populations with natural diets (Coogan & Raubenheimer, 2016).

## 2 MATERIALS AND METHODS

### 2.1 Macronutrient composition of diets

We started with studies collected from a global review of brown bear diets (Bojarska & Selva, 2012). We required estimates of the dietary proportion of mass of food consumed to calculate macronutrient compositions; thus, we included studies where foods were originally reported as the proportion of dry mass of diet (digestible dry matter; %DDM) or were estimable after applying fecal correction factors (CF) to percent fecal volume (%Vol) estimates. We updated our search to find studies published between 2012 and 2017 using Google Scholar and the search term *brown bear diet*, as well as searching within literature citing the aforementioned review. Other articles were obtained via ResearchGate (www.researchgate.net). We excluded studies where: (1) food categories were considered too broad to reasonably estimate the nutritional composition of the diet; (2) did not provide %Vol or %DDM estimates of diet; (3) did not cover the brown bear active season; and (4) there were imbalances in sampling that resulted in overestimating season‐specific food resources. All studies exceeded the lowest scat sample size (*n* = 95) cited in Bojarska and Selva (2012). This left us with a total of 18 papers providing data for 19 “populations” (Table 1). Populations were considered independent if samples were taken from different studies, countries, geographic regions, habitats, or years, following Senior et al. (2016).

**Table 1:** Selected studies of brown bear diets

Diet_ID	References	Country
1	MacHutchon & Wellwood (2003)	Canada
2	Gau et al. (2002)	Canada
3	Munro et al. (2006)	Canada
4	McLellan & Hovey (1995)	Canada
5	Mattson et al. (1991)	USA
6	Clevenger et al. (1992)	Spain
7	Naves et al. (2006)	Spain
8	Dahle et al. (1998)	Sweden
9	Dahle et al. (1998)	Norway
10	Persson et al. (2001)	Norway
11	Rigg & Gorman (2005)	Slovakia
12	Vulla et al. (2009)	Estonia
13	Sato et al. (2004)	Japan
14	Stenset et al. (2016)	Sweden
15	Ciucci et al. (2014)	Italy
16	Paralikidis et al. (2010)	Greece
17	Fortin et al. (2013)	USA
18	Kavčič et al. (2015)	Slovenia
19	Stofik et al. (2013)	Slovakia

The use of CFs is considered among the most suitable methods for brown bear diet assessment (Bojarska & Selva, 2012); however, they can result in variable outcomes depending upon their application, particularly for ungulates (López‐Alfaro et al., 2015; Persson, Wikan, Swenson, & Mysterud, 2001). Thus, where possible, we used %Vol estimates given in cited papers and applied our chosen CFs to re‐estimate the %DDM of foods in diets. We chose this approach to standardize the CFs used thereby minimizing diet variation across studies due to their differential application. For papers that gave seasonal estimates, we estimated %DDM for each season and from this we estimated the annual diet. We considered each seasonal diet to be representative for that time period, as opposed to weighting by sample size, to avoid biasing annual diets toward seasonal food items where sample size was not evenly distributed. For analysis of seasonal diets, we classified seasonal diet estimates as being one of four categories: spring; summer; autumn; or winter. We used the CFs presented in Hewitt and Robbins (1996), as applied by Fortin et al. (2013) and López‐Alfaro et al. (2015) to different food categories. We also applied CFs where available to specific food items within soft mast, hard mast, insects, and small mammal categories following Hewitt and Robbins (1996) and Bojarska and Selva (2012). In brief, CFs are applied by multiplying %Vol estimates of food items by their respective CF (i.e., %Vol * CF). These values are then summed across food items, and the %Vol * CF for each food item is expressed as a percentage of this sum to yield %DDM estimates (see Table 2 in Hewitt & Robbins, 1996). CFs are given in Table S1.

**Table 2:** (A) Matrix containing the geometric mean pairwise ratios of macronutrients in annual bear diets; and (B) variance matrix of log‐ratios among macronutrients in annual bear diets

(A) Mean ratio matrix			
	P	C	L
P	1	0.906	0.928
C	1.104	1	1.024
L	1.078	0.976	1
(B) Variance matrix			
	P	C	L
P	0	0.700	0.197
C	0.700	0	0.577
L	0.197	0.577	0

After estimating %DDM of food items in diets, we estimated the macronutrient composition of each food or food group using data collected from the literature and the USDA National Nutrient Database (US Department of Agriculture 2015; Table S1). Graminoids and forbs were condensed into one food category each. For other food categories, we obtained species‐specific food estimates or proxies, where possible. For animal prey, we used estimates of whole carcasses, because estimates of only muscle tissue likely overestimate protein and underestimate lipid content (Coogan et al., 2014), and brown bears tend to eat entire carcasses (Hilderbrand, Jenkins, Schwartz, Hanley, & Robbins, 1999). When possible we used total dietary fiber (TDF) estimates of indigestible carbohydrates to avoid differences in available carbohydrate estimates by subtraction and to more closely match the digestibility of bears (Pritchard & Robbins, 1990). Macronutrients were converted to percent metabolizable energy (Coogan et al., 2014, 2017) using conversion factors of 17 kJ/g for protein and carbohydrate and 37 kJ/g for lipid (Merrill & Watt, 1973). The proportions of macronutrient energy in foods were weighted by %DDM estimates to estimate their overall proportions in diets.

We were unable to obtain nutritional composition estimates of reported foods that were spatially and temporally contemporary with bear fecal samples in the published studies, which may induce error in macronutrient estimates of certain foods. This approach, however, is unlikely to significantly affect comparisons of macronutrient proportions between populations (Remonti et al., 2016; Senior et al., 2016).

### 2.2 Data analysis

We used graphical devices and associated theory from nutritional geometry to inform our analysis of macronutrient proportions in bear diets. Nutritional geometry is a multivariate graphical approach to examining nutrition based on state–space models and has been applied to a variety of species in both laboratory and free‐ranging settings (Raubenheimer et al., 2015; Simpson & Raubenheimer, 2012). Because our data set was compositional (i.e., a vector of non‐negative components which sum to a constant) and consisted of three components, we plotted bear diets within a simplex using mixture triangles (Raubenheimer, 2011). Specifically, we used conventional ternary diagrams, or equilateral mixture triangles (EMT), to visualize and interpret data. We provide right‐angled mixture triangle (RMT) plots for comparison (Figures S1 and S2). For information on the use of mixture triangles in the context of nutritional ecology, we refer the reader to Raubenheimer (2011).

We used compositional data analysis to analyze the proportions of macronutrients in bear diets. Compositional data analysis is a field of statistics that was developed to address concerns regarding using conventional statistics to analyze compositional data (Aitchison, 1982) and has been used across a variety of fields, including geosciences (Buccianti, Nisi, Martín‐Ferández, & Palarea‐Albaladejo, 2014), public health (Chastin, Palarea‐Albaladejo, Dontje, & Skelton, 2015), and meat science (Ros‐Freixedes & Estany, 2013). A full review of compositional data analysis is beyond the scope of this study; hence, we refer readers to the papers cited herein.

We used the R (v.3.4.1; R Core Team 2017) package {compositions} (v.1.40‐1; van den Boogaart, Tolosana, & Bren, 2014) for our compositional analysis in acomp geometry (van den Boogaart & Tolosana‐Delgado, 2008). We first examined annual diets, where we reported compositional descriptive statistics and variance for annual diets as the closed geometric mean and variance matrix of our centered log‐ratio (clr) transformed data set. The compositional geometric mean better represents the center of compositional data points than the arithmetic mean, and dispersion of compositional data is summarized using a variance matrix of pairwise log‐ratios (Aitchison, 2003). Conventional univariate measures of dispersion (e.g., *SD* of the arithmetic mean proportion of protein) are not considered informative for multivariate compositional data. For comparison, however, we report both conventional arithmetic and geometric means. We plotted predicted 2‐sigma and 3‐sigma region ellipsoids around the geometric mean.

We used a principal component analysis in acomp geometry (PCA.acomp) to examine variance in the proportions of macronutrients in annual population diets (Aitchison, 1983; Aitchison & Greenacre, 2002; Pawlowsky‐Glahn & Egozcue, 2001). PCA.acomp axes were plotted both within an EMT as curvilinear axes and using a biplot. In PCA.acomp biplots, the length of the link (i.e., distance between arrowheads) along a component relates to the *SD* of the log‐ratio of two components. Thus, the distance between links is used to evaluate relative variation between components.

To examine differences between seasons, we used linear models (LM) to examine changes in the proportion of macronutrients in bear diets using an isometric log‐ratio (ilr) data transformation following Tolosana‐Delgado and van den Boogaart (2011). The ilr transformation adjusts for changes in the proportion of one macronutrient consumed with the proportion of others consumed and allows for the use of conventional statistics on the transformed data, which is then back transformed into the original units for interpretation. Season was set as an ordered 3‐level categorical variable (spring, summer, and autumn), with spring set as the intercept category. Winter (*n* = 3) observations were dropped from the statistical analysis. Differences in seasonal diets were evaluated statistically in the global LM using an ANOVA. Model residuals were assessed for normality and homoscedasticity. Differences between individual seasons were assessed graphically by plotting geometric means and both 90% and 99% confidence regions within an EMT. For comparison with our compositional model, we created three separate univariate LMs of the effect of season (as an ordered factor) on the logit‐transformed (Warton & Hui, 2011) decimal proportion of each macronutrient. We followed the same ilr approach to examine differences in the annual and seasonal diets of populations receiving anthropogenic “subsidies” (e.g., agricultural crops, livestock, and supplemental feeding) versus natural diets (set as a binary explanatory variable).

To examine diets in relation to the behavioral preferences of captive bears, we plotted the mean proportion of protein (17%) as an isoportion line (“intake target” sensu Simpson & Raubenheimer, 2012) within EMTs. The preferred optimal ratio of macronutrients is likely to vary between bears (Erlenbach et al., 2014) and perhaps populations (Shafer et al., 2014); thus, we also plotted the associated ±4% *SD* isoportion lines around the mean protein intake to represent variance. We note as caveats that the preferred mean protein intake of captive bears was determined using conventional statistics which might differ from the compositional mean. Likewise, as mentioned previously, conventional *SD* estimates are not consistent with a compositional data analysis paradigm. Nonetheless, given the difficulty in determining macronutrient intake targets and related functional outcomes of free‐ranging animals (Machovsky‐Capuska, Coogan, Simpson, & Raubenheimer, 2016), adopting the optimal diet reference point of captive bears serves as a useful heuristic.

## 3 RESULTS

### 3.1 Annual diets

**Figure 1:**
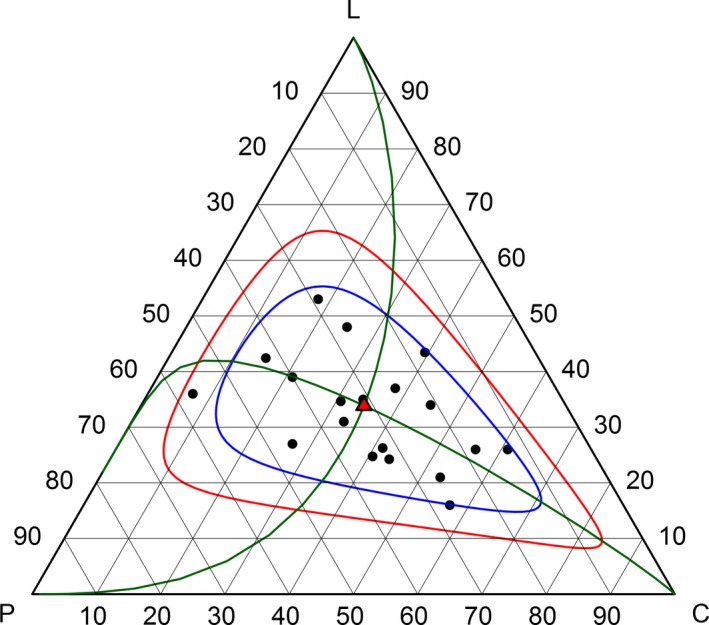
Equilateral mixture triangle (EMT) depicting the proportions of macronutrients (protein = P, carbohydrate = C, and lipid = L) in annual bear diets (black dots). The geometric mean is shown by a red triangle. Ellipsoids predicting 2‐sigma and 3‐sigma regions are given in blue and red, respectively. Curvilinear principal component axes in acomp geometry (PCA.acomp) are shown with green lines. Component 1 (“horizontal” curve) explained 80.2% of the variance, and component 2 (“vertical” curve) explained 19.2% of variance. Corners represent 100% composition of the labeled macronutrient

Across annual diets, the closed geometric mean proportion of macronutrient energy was 31.4% protein, 34.7% carbohydrate, and 33.9% lipid, which lies near to the mixture triangle's barycentre (Figure 1). The arithmetic mean (±*SD*) proportions of macronutrients were as follows: 31.0% (±10.7) protein; 36.1% (±14.9) carbohydrate; and 32.8% (±9.5) lipid. Thus, differences between the compositional and arithmetic means were relatively small, being 1.4% for carbohydrate, 1.1% for lipid, and 0.4% for protein.

Variability in the proportion of macronutrients in population diets is summarized in the variance matrix containing the pairwise log‐ratio variances (Table 2). Values close to zero indicate that the macronutrients in the ratio are highly proportional/codependent (i.e., relatively more constant). Protein and lipid in bear diets have log‐ratio variance closest to zero, implying that there is a higher proportional relationship between the consumption of the two macronutrients. Conversely, the highest log‐ratio variances occur with carbohydrate, which indicates that carbohydrate in bear diets is the least codependent on the other macronutrients. Following the 68‐95‐99.7 rule, ca. 95% and 99% of values are predicted to lie within 2 and 3 standard deviations of the mean; thus, the 2‐sigma and 3‐sigma regions in Figure 1 can serve as an estimate of the fundamental macronutrient niche of brown bears.

**Figure 2:**
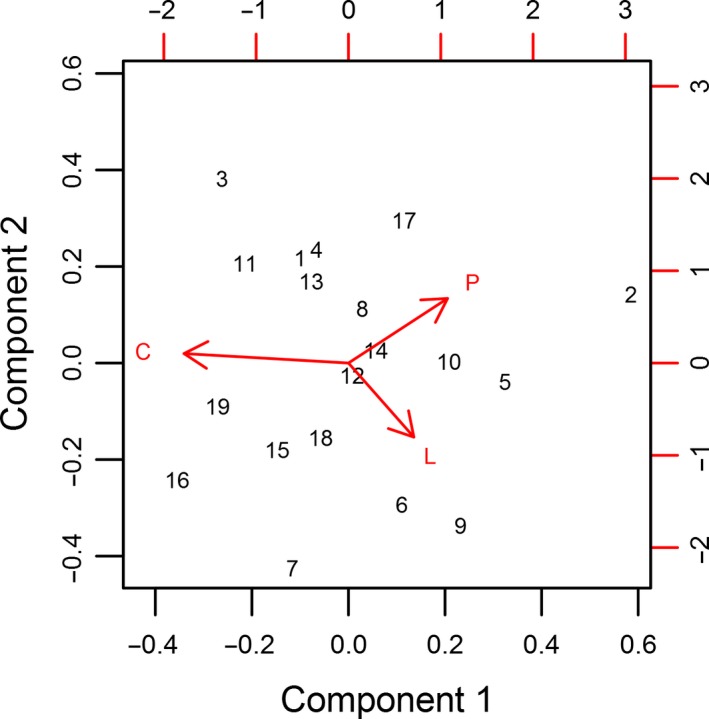
Principal component analysis in Aitchison geometry (PCA.acomp) biplot of the proportion of macronutrients (protein = P, carbohydrate = C, and lipid = L) in annual brown bear diets. Numbers correspond to populations in Table 1. The relevant variables in the PCA.acomp biplot are the “links” (i.e., difference between two arrowheads) which represents the *SD* of log‐ratios between two components. Thus, the greatest relative variation occurred among protein and carbohydrate ratios, while the ratios of protein and lipid were relatively more proportional

The first component of the PCA.acomp, associated with differences in ratios of carbohydrate with both lipid and protein, explained 80.8% of variance in the macronutrient proportions of bear diets (Figures 1 and 2). The remaining second component, associated with lipid and protein ratios, explained the remaining 19.2% of variance. In the PCA.acomp biplot (Figure 2), the link distance between protein and carbohydrate was greatest, indicating that most relative variation occurs between these two macronutrients. Carbohydrate and lipid also share a large amount of relative variation. The shorter link between protein and lipid indicates that their component ratio was relatively more constant.

### 3.2 Anthropogenic subsidies

**Figure 3:**
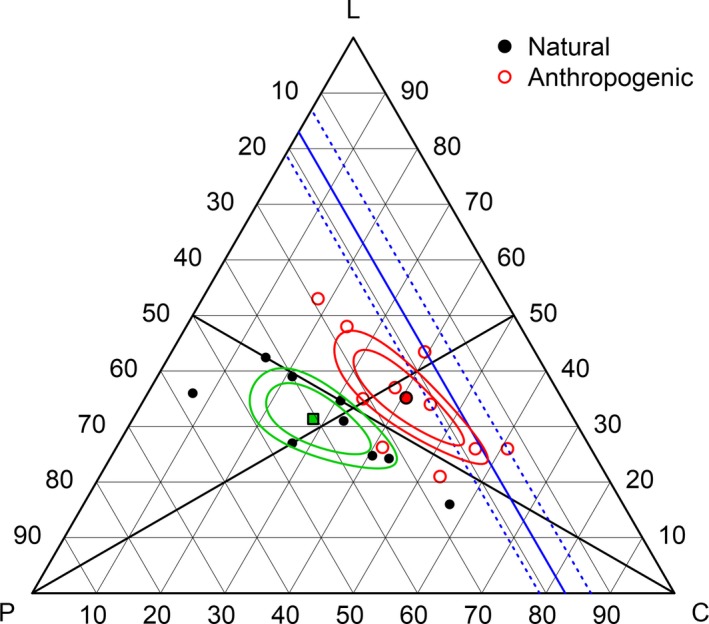
EMT showing differences between the annual diets of bear populations receiving anthropogenic subsidies versus natural diets. Means (filled symbols) are shown with 90% and 99% confidence regions. The blue line represents the preferred optimal proportion of protein (17% ± 4) selected by captive bears. Isoproportion lines represent 1:1 proportions of protein and lipid (radiating from the 100% carbohydrate corner) and carbohydrate and lipid (radiating from the 100% protein corner)

Annual diets with anthropogenic subsidies were significantly different to those with natural foods based on an arbitrary α = 0.05 (ANOVA *p* < .001; Figure 3). The geometric mean percentage of macronutrients in diets with anthropogenic subsidies was 24.2% protein, 40.6% carbohydrate, and 35.2% lipid. For natural diets, mean proportions were 40.5% protein, 28.1% carbohydrate, and 31.4% lipid. The 99% confidence region of the mean anthropogenic diet included the isoportion line representing the preferences of captive bears, suggesting that such an annual diet is possible for bears consuming anthropogenic subsidies. The closer alignment of geometric means along the 1:1 isoproportion line for lipid and carbohydrate (radiating from the 100% protein corner) indicates a stronger decrease in protein relative to the other macronutrients. The separation of means and confidence regions between the 1:1 isoproportion line for protein and lipid (radiating from the 100% carbohydrate corner) shows that the ratio of lipid relative to protein is higher in anthropogenically subsidized populations. The shape of the confidence regions around means shows that there is more variation in carbohydrate in both groups.

### 3.3 Seasonal diets

**Figure 4:**
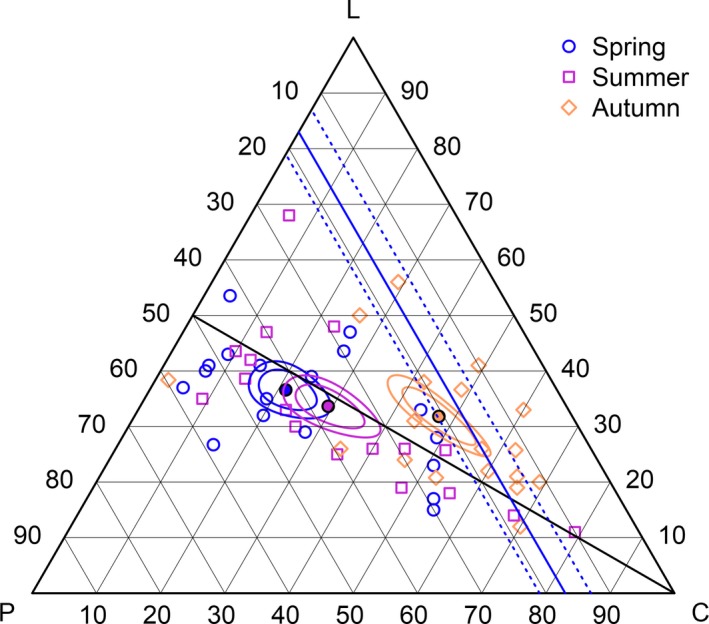
EMT of the proportions of macronutrients (protein = P, carbohydrate = C, and lipid = L) in seasonal brown bear diets. The geometric mean for each season is shown by a filled symbol surrounded by 90% and 99% confidence regions. For reference, the blue line represents the preferred optimal proportion of protein (17% ± 4) selected by captive bears. A black isoproportion line represents 1:1 proportions of protein and lipid

The proportions of macronutrients in all bear diets varied significantly between seasons (ANOVA *p* < .001). Comparing spring to autumn, the geometric mean proportion of macronutrients in diets declined in protein (20.4%) and lipid (4.8%) and increased in carbohydrate (26.2%) (Table 3). Both spring and summer show overlapping confidence regions around mean compositions, suggesting they are not different (Figure 4). Autumn diets, however, lie distinctly in a higher carbohydrate, lower protein region of the simplex. Conventional univariate models were in agreement with compositional analysis: Protein showed a statistically significant linear decrease, and carbohydrate showed a significant increase, from spring to autumn (Table S4). Within‐season variability (i.e., interpopulation) in the proportion of macronutrients in population diets is summarized in the variance matrix in Table 3. For all seasons, protein and lipid had the highest codependence, with carbohydrate being the least codependent on the other macronutrients.

**Table 3:** (A) Geometric mean decimal proportion of protein (P), carbohydrate (C), and lipid (L) in seasonal bear diets across all populations and partitioned into those receiving anthropogenic subsidies versus natural diets. (B) Matrix containing the geometric mean pairwise ratios of macronutrients in seasonal diets. (C) Variance matrix of log‐ratios among macronutrients in seasonal diets

	Spring			Summer			Autumn		
(A) Geometric mean	P	C	L	P	C	L	P	C	L
Combined	0.422	0.212	0.366	0.371	0.293	0.336	0.208	0.474	0.318
Natural	0.493	0.159	0.348	0.447	0.229	0.325	0.293	0.408	0.300
Anthropogenic	0.351	0.275	0.374	0.290	0.372	0.338	0.142	0.532	0.326
(B) Mean ratio matrix									
Combined									
	P	C	L	P	C	L	P	C	L
P	1.000	1.993	1.154	1.000	1.267	1.102	1.000	0.438	0.652
C	0.502	1.000	0.579	0.789	1.000	0.870	2.283	1.000	1.490
L	0.867	1.727	1.000	0.907	1.149	1.000	1.533	0.671	1.000
Natural									
	P	C	L	P	C	L	P	C	L
P	1.000	3.105	1.419	1.000	1.951	1.376	1.000	0.718	0.975
C	0.322	1.000	0.457	0.513	1.000	0.705	1.393	1.000	1.358
L	0.705	2.187	1.000	0.727	1.418	1.000	1.026	0.736	1.000
Anthropogenic									
	P	C	L	P	C	L	P	C	L
P	1.000	1.279	0.938	1.000	0.780	0.859	1.000	0.267	0.437
C	0.782	1.000	0.733	1.283	1.000	1.102	3.742	1.000	1.634
L	1.066	1.364	1.000	1.164	0.908	1.000	2.290	0.612	1.000
(C) Variance matrix									
Combined									
	P	C	L	P	C	L	P	C	L
P	0.000	1.239	0.144	0.000	1.230	0.149	0.000	1.478	0.394
C	1.239	0.000	1.220	1.230	0.000	1.499	1.478	0.000	1.089
L	0.144	1.220	0.000	0.149	1.499	0.000	0.394	1.089	0.000
Natural									
	P	C	L	P	C	L	P	C	L
P	0.000	1.250	0.065	0.000	0.778	0.031	0.000	2.377	0.217
C	1.250	0.000	1.375	0.778	0.000	0.972	2.377	0.000	1.958
L	0.065	1.375	0.000	0.031	0.972	0.000	0.217	1.958	0.000
Anthropogenic									
	P	C	L	P	C	L	P	C	L
P	0.000	0.941	0.145	0.000	1.414	0.170	0.000	0.214	0.256
C	0.941	0.000	1.093	1.414	0.000	2.195	0.214	0.000	0.337
L	0.145	1.093	0.000	0.170	2.195	0.000	0.256	0.337	0.000

**Figure 5:**
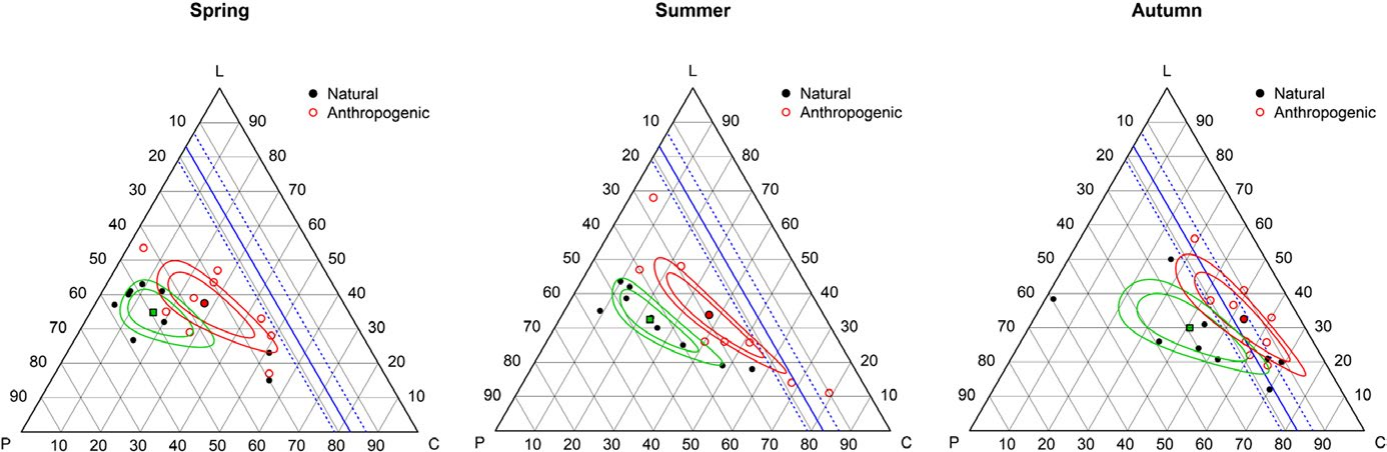
EMT of the proportions of macronutrients (protein = P, carbohydrate = C, and lipid = L) in seasonal (spring, summer, and autumn) brown bear diets in populations with natural diets versus those receiving anthropogenic subsidies. The geometric mean for each season is shown by a filled symbol surrounded by 90% and 99% confidence regions. For reference, the blue line represents the preferred optimal proportion of protein (17% ± 4) selected by captive bears

However, intraseasonal differences in mean macronutrient proportions were revealed when comparing between anthropogenic and natural diets (Spring ANOVA *p* = .019; Summer ANOVA *p* < .001; and Autumn ANOVA *p* = .012) (Table 3; Figure 5). Across seasons, diets receiving anthropogenic subsidies tended to be higher in carbohydrate and lower in protein than natural diets.

Seasonally, many diets were close to the 17% isoportion line during autumn (Figure 4). The 99% confidence region around the autumn geometric mean proportion included the 17% protein isoportion line, which suggests they are not significantly different at that level. Both spring and summer diets were generally higher than 17% protein. Of the three winter diets reported, two were near the 17% line, while one was noticeably lower (not shown). During autumn, mean diets of both anthropogenic and natural diets had confidence intervals including the intake target region of captive bears (Figure 5); however, diets receiving anthropogenic subsidies made up the majority of autumn diets near to the intake target.

There was considerably more variation in the proportion of protein and carbohydrate consumed in natural compared to anthropogenic diets during autumn, with one population (Gau et al. (2002), Diet_ID: 2; Table 1) consuming very little (2%) carbohydrate. Because this natural diet was a potentially influential observation (as assessed visually in R using a plot of the cooks.distance (Cook & Weisberg, 1982) function in the {base} package), we performed a sensitivity analysis by running a separate LM and ANOVA for autumn without that diet. Anthropogenic and natural diets remained significantly different (ANOVA *p* = .021) in autumn after removal of the Gau, Case, Penner, and McLoughlin (2002) diet, albeit with a lower mean proportion of protein and lipid and higher proportion of carbohydrate (P = 0.232, C = 0.515, L = 0.253)—thus, the mean autumn diet of natural populations was closer to the intake target region with that diet removed.

A greater number of anthropogenic diets were higher in carbohydrate and lower in protein than the intake target; conversely, there were a greater number of natural diets higher in protein and lower in carbohydrate relative to the intake target of captive bears. Of note, confidence regions around the mean summer anthropogenic diet included the upper end of the intake target region for protein, while an individual diet point lied near to the 17% target line, indicating that the preferred ratio self‐selected by captive bears is also achievable during summer for some bear populations consuming human‐sourced foods.

## 4 DISCUSSION

The range of dietary macronutrient proportions that we observed among brown bear populations supports the nutritional generalism hypothesis that the species has a wide fundamental macronutrient niche. In combination with previous studies documenting the types and compositions of foods consumed by this species, the brown bear can thus be classified as generalist in all three aspects of the multidimensional nutritional niche. Across populations, the geometric mean annual diet of brown bears was close to an equal one‐third proportion (i.e., the simplex barycentre) for all macronutrients despite considerable interpopulation variance, suggesting that the species has a remarkable ability to tolerate the macronutritional characteristics of their nutritional environment. Thus, we provide evidence that one function of omnivory in brown bear is to enable occupation of a diverse range of habitats and macronutritional environments.

Annual diet variation in macronutrient proportions was not equal; however, as across populations, the greatest variation was observed for carbohydrate, while protein and lipid were more codependent. The nutritional explanation for this is that animal prey is a source of both protein and lipid, with negligible carbohydrate content (Coogan et al., 2014). The highest proportion of protein in annual diets, and lowest proportion of carbohydrate, was found in Canada's central Arctic, where bears displayed high levels of predation on caribou (*Rangifer tarandus*) and ground squirrels (*Spermophilus parryii*; Gau et al., 2002). Diets relatively high in carbohydrate occurred in ecosystems where bears consumed starchy roots (e.g., *Hedysarum alpinum*) and fruit (e.g., Munro, Nielsen, Price, Stenhouse, & Boyce, 2006). However, carbohydrate proportions were highest in annual diets with anthropogenic subsidies, such as agricultural crops and supplemental feeding of corn, oats, and wheat (e.g., Paralikidis, Papageorgiou, Kontsiotis, & Tsiompanoudis, 2010; Rigg & Gorman, 2005; Sato, Aoi, Kaji, & Takatsuki, 2004; Stofik, Merganic, Merganicova, & Saniga, 2013). Similarly, the proportion of lipid in annual diets was highest in populations that consumed relatively more domestic livestock (e.g., Clevenger, Purroy, & Pelton, 1992; Dahle, Sorensen, Wedul, Swenson, & Sandergren, 1998). Therefore, our results support the hypothesis that bear diets including anthropogenic food subsidies are, on average, higher in proportions of nonprotein macronutrients, especially carbohydrate.

Seasonally, brown bear displayed significant variation in the proportion of macronutrients consumed, indicating a tolerance for a wide range of dietary macronutrient proportions throughout the active season thereby supporting our third hypothesis. Protein and lipid proportions become less codependent during autumn, which is consistent with the general pattern of decreasing carnivory combined with the consumption of high‐fat autumn food resources in some ecosystems, such as hard mast. The proportion of carbohydrate in bear diets was highest in the autumn, mostly due to the timing of fruit production, while some populations also consumed starchy roots during this period. However, diets of populations receiving anthropogenic subsidies were on average higher in the proportion of carbohydrates and lower in protein, across all seasons. For example, the diets of Greek (Paralikidis et al., 2010), Italian (Ciucci et al., 2014), and Slovakian (Stofik et al., 2013) bears were high in carbohydrate during summer, due to fruit and anthropogenic food consumption. Given that such foods allow bears to consume closer to preferred proportions of macronutrients, it is not surprising that anthropogenic foods are sources of bear–human conflict (Can, D'Cruze, Garshelis, Beecham, & Macdonald, 2014; Coogan & Raubenheimer, 2016; Morehouse & Boyce, 2017). Garbage, which was not considered in this study and seldom reported (e.g., Mattson, Blanchard, & Knight, 1991; Rigg & Gorman, 2005), would likely have a similar effect on diet proportions (Coogan & Raubenheimer, 2016). Conversely, the highly carnivorous natural diet of central Arctic bears (Gau et al., 2002) was highest in protein during the autumn.

The mean proportions of macronutrients consumed by bears in autumn were generally near that self‐selected by captive bears, which supports our hypothesis that the optimal diet preferences of bears coincide with the nutritional environment during the hyperphagic period due to the strong selective pressures associated with hibernation. For instance, there is a strong relationship between the body fat percentages of bears and their survival and reproductive capacity during hibernation (López‐Alfaro et al., 2013; Robbins, Meray, Fortin, & Lynne Nelson, 2012). In addition to behavioral adaptation, brown bears have acquired a suite of physiological adaptations facilitating adiposity while simultaneously remaining healthy (Rivet, Nelson, Vella, Jansen, & Robbins, 2017). Bears primarily gain lean mass, if any, during the spring season when their diets are higher in protein content (Hilderbrand et al., 1999; Swenson, Adamic, Huber, & Stokke, 2007). Yet, the importance of spring lean mass accrual should not be underestimated, as protein is transferred from mother to cub via milk during the hibernation period (López‐Alfaro et al., 2013).

Relative to natural diets, however, bears receiving anthropogenic subsidies were closer to the intake target of captive bears during autumn. Furthermore, there was less variation in autumn anthropogenic diets relative to the intake target region, which suggests that bears in such populations are not only more likely to consume optimal diets but are also buffered from environmental limitations in natural food supply. Thus, brown bears receiving anthropogenic subsidies as part of their diets may have a nutritional advantage over those consuming natural diets. However, bears consuming anthropogenic subsidies may also be more likely than natural populations to consume lower proportions of protein than optimal which may adversely affect fitness outcomes. For example, diets lower in protein than the preferred proportion selected by captive bears resulted in lower rates and efficiency of gain compared to diets higher in protein than the self‐selected optima (Erlenbach et al., 2014).

As mentioned, there was noticeable variation among macronutrient proportions of populations with natural diets, with some noticeably higher in the proportion of protein selected by captive bears. Given that the dietary preferences and optima are expected to be under natural selection, it is thus possible that the intake target of brown bear varies among populations. For example, populations consuming high proportions of protein and very little carbohydrate, such as an in the central Arctic (Gau et al., 2002), may have different intake targets than populations which have evolved under different environmental conditions—even within populations, marked differences in individual foraging behavior (i.e., carnivory versus herbivory) have been observed (Edwards, Derocher, Hobson, Branigan, & Nagy, 2011). Likewise, such dietary adaptation has implications for populations receiving anthropogenic subsidies if their dietary optima shift in response to their nutritional environment, especially if such subsidies no longer become available. At the same time, however, the range of macronutrient proportions observed across populations of this species demonstrates their remarkable tolerance to varying dietary macronutrient proportions.

The wide multidimensional nutritional niche of brown bear supports previous suggestions that, as a species, brown bears may be better equipped to face some of the nutritional challenges associated with climate change, such as changes in available food resources (Roberts, Nielsen, & Stenhouse, 2014). Yet, there may be unexpected relationships between brown bears and changing climate, as their macronutrient preferences may have broad ecological implications when the timing of seasonal food resources changes. One study, for example, found that brown bears preferentially switched to eating fruit that became available several weeks early in place of available spawning salmon they historically consumed during that period (Deacy et al., 2017). Brown bear's preference for high proportions of nonprotein macronutrients was given as a possible explanation for this diet shift (i.e., the proportion of macronutrients in some fruit is very close to the preferred ratio of captive bears; Coogan et al., 2014). This situation is similar to the case of bears receiving anthropogenic subsidies, in that they are able to feed on foods offering macronutritional properties otherwise temporally or ecologically unavailable.

An interesting extension of this research is to explore how dietary macronutrient proportions influence the fitness and population demographics of brown bear. Macronutrient proportions have physiological effects on individual body composition, with high‐protein diets generally resulting in animals with lower body fat and greater lean mass than animals on high‐carbohydrate or ‐lipid diets and vice versa (Solon‐Biet et al., 2014). This pattern can be observed among brown bears, which gain primarily lean mass in spring and fat mass in autumn. Examining other effects of macronutrient proportions on bear populations may be revealing. Low‐protein, high‐carbohydrate diets have been associated with increased longevity and health span across several model organisms; conversely, high‐protein, low‐carbohydrate diets have been associated with reduced lifespan, but increased reproductive parameters (Raubenheimer, Simpson, Le Couteur, Solon‐Biet, & Coogan, 2016).

Furthermore, there is implicit evidence that proportions and amounts of dietary macronutrients interact to affect brown bear population dynamics, as local population density has been related to spatial patterns in the amounts of both ungulates (source of protein and lipid) and fruit (source of carbohydrate) together (Nielsen, Larsen, Stenhouse, & Coogan, 2017). Following the above example, it is important to note that both the proportions and amounts of macronutrients interact to produce biological outcomes; therefore, investigating the relationships between the amounts and proportions of dietary macronutrients, and their possible population‐level effects, is an important area of future research. On the other hand, in many animals, dietary macronutrient proportions predict absolute amounts eaten (Raubenheimer, Machovsky‐Capuska, Gosby, & Simpson, 2014).

Leading from this, we propose that future research examine spatially explicit factors influencing the macronutrient proportions of diet. Increasing carnivory has been hypothesized as a general adaptation to an increase in latitude in omnivorous mammals (Vulla et al., 2009); however, other works have suggested that dietary patterns are better explained by spatially explicit environmental factors (Gaston, Chown, & Evans, 2008). Patterns in brown bear diet were better explained by climatic rather than geographic factors (Bojarska & Selva, 2012). It would be interesting to examine the relationships between such factors and nutrition. Furthermore, we suggest the effects of anthropogenic food subsidies on brown bears at the levels of individuals, populations, and communities deserve more research.

In closing, we present a synthesis of macronutritional niche theory, nutritional geometry, and compositional analysis to produce a novel view of the nutritional ecology of brown bear and functional omnivory more generally. Furthermore, we demonstrate the effect of anthropogenic subsidies on the macronutrient proportions of brown bear diet, and the implications of which are open to future study. Last, while it may be argued that compositional analysis is the appropriate way to analyze proportional data, our univariate tests were in agreement with compositional results. Similar results between these methods have been documented elsewhere, where it was suggested that traditional statistical methods are robust to compositions if variance is not too great (Ros‐Freixedes & Estany, 2013).

## CONFLICT OF INTEREST

None declared.

## AUTHORS' CONTRIBUTIONS

SCPC and DR conceived the original idea. SCPC designed the methodology, collected and analyzed the data, and wrote the first draft. DR, GBS, NCC, SEN, and SCPC critically reviewed and revised the manuscript.

## DATA ACCESSIBILITY

Data and sources used herein are provided in the Supporting Information.
