# Supplementary material for: Functional macronutritional generalism in a large omnivore, the brown bear
Source: Ecol Evol. 2018 Jan 29;8(4):2365–76. doi: 10.1002/ece3.3867 (PMC5817158; doi:10.1002/ece3.3867)
Supplement: Supplementary file 4 [file ECE3-8-2365-s004.pdf]

Supplemental information

Table S4. Results of univariate linear models of the logit-transformed decimal proportions of macronutrients in bear diets as a function of season (spring, summer, autumn) as an ordered factor.

Macronutrient	Estimate	Std. Error	t value	$Pr(>\lvert t\rvert)$
Protein				
(Intercept)	-0.85628	0.08381	-10.217	7.78E-14
season.L	-0.7153	0.14377	-4.975	8.09E-06
season.Q	-0.21093	0.14656	-1.439	0.156
Carbohydrate				
(Intercept)	-0.79	0.1529	-5.168	4.16E-06
season.L	0.8388	0.2622	3.199	0.0024
season.Q	0.131	0.2673	0.49	0.6263
Lipid				
(Intercept)	-0.79271	0.08099	-9.788	3.29E-13
season.L	-0.16096	0.13892	-1.159	0.252
season.Q	0.01909	0.14162	0.135	0.893

RMT plots

Here we used the more recently developed right-angled mixture triangle (RMT; Raubenheimer 2011) to examine the macronutrient proportions of brown bear diets. The RMT differs from the EMT in that it partitions simplex dimensions into focal (*x* and y) and implicit (z) axes. Captive bears prioritized constant protein intake (17%), with variable carbohydrate and lipid intake; thus, we set the focal RMT axes to represent carbohydrate (x-axis) and lipid (y-axis), with protein represented on the implicit axis. We placed a convex hull around annual diet points in the RMT, to outline an estimate of the fundamental macronutrient niche of brown bears following Machovsky-Capuska et al. (2016a) and Senior et al. (2016).

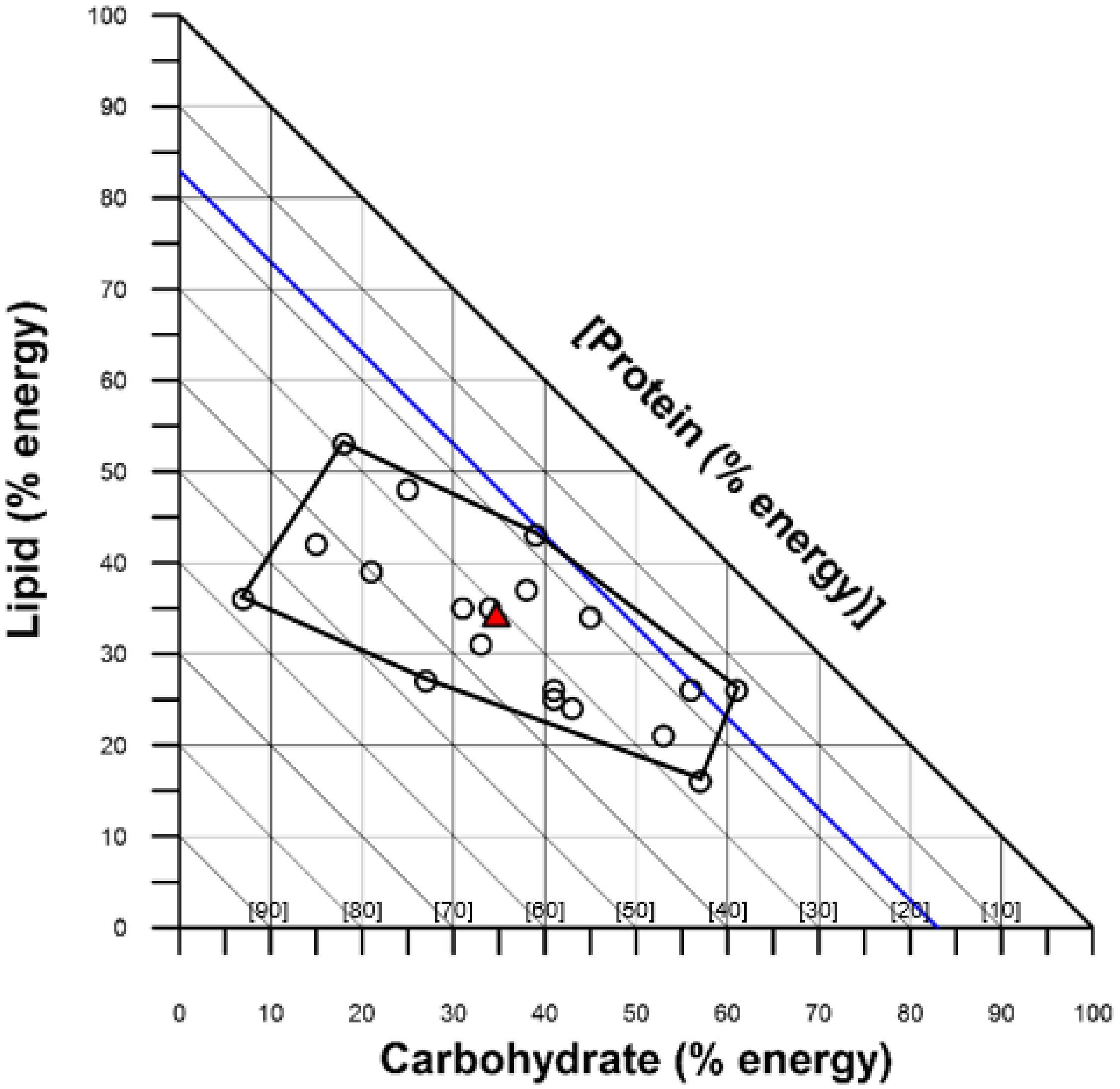

Fig. S1. Right-angled mixture triangle (RMT; Raubenheimer 2011) of annual brown bear diets worldwide, highlighting the relationship between carbohydrate and lipid (i.e. the non-protein macronutrients) on the focal *x* and *y* axes, and protein on the implicit *z* axis. The geometric mean diet is plotted as the red triangle. For reference, the preferred arithmetic mean proportion of protein to non-protein macronutrient energy selected by captive brown bears (Erlenbach *et al*. 2014) is shown as the blue diagonal isoportion line at 17% protein energy. A convex hull is placed around the diet points, outlining a conservative estimate of the fundamental macronutrient niche of brown bears.

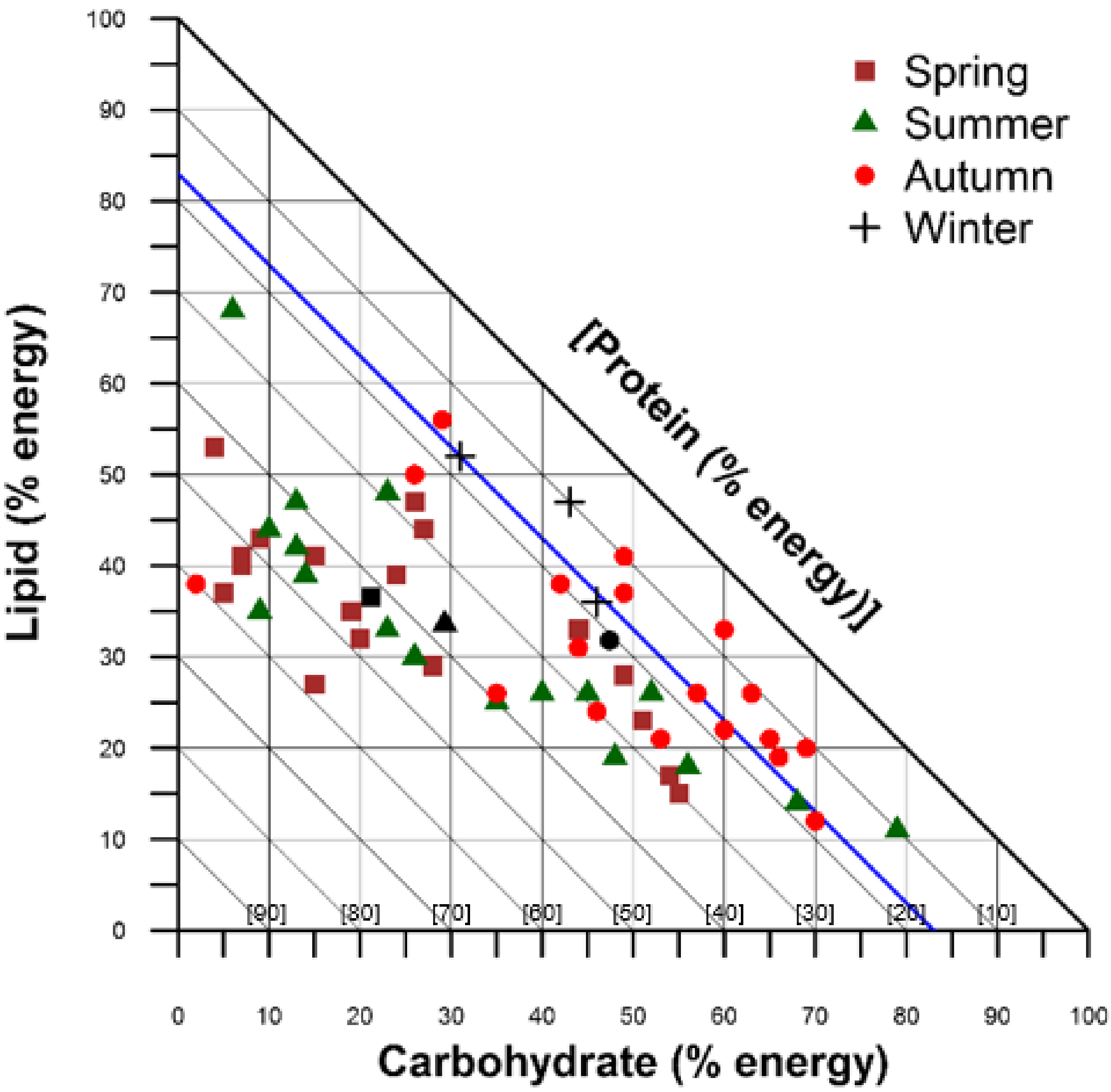

Fig. S2. RMT of seasonal brown bear diets highlighting the relationship between carbohydrate and lipid (i.e. the non-protein macronutrients) on the focal *x* and *y* axes, and protein on the implicit *z* axis. Seasonal geometric mean proportions are shown in black. The preferred proportion of protein to non-protein macronutrient energy selected by captive brown bears is shown as the blue isoportion line at 17% protein. The winter season was withheld from statistical analysis, but is shown here for comparison.

Bibliography of references used for macronutrient composition of brown bear foods in Table S1.

Alcantara, R.M., Hurtada, W.A, & Dizon, E.I. 2013. (2013) The nutritional value and phytochemical components of taro [*Colocasia esculenta* (L.) Schott] powder and its selected processed foods. *Journal of Nutrition & Food Sciences*, **3**, 207.

Colomer-Rocher, F., Kirton, A.H., Mercer, G.J.K. & Duganzich, D.M. (1992) Carcass composition of New Zealand Saanen goats slaughtered at different weights. *Small Ruminant Research*, **7**, 161-173.

Cook, R.C., Cook, J.G., Murray, D.L., Zager, P., Johnson, B.K. & Gratson, M.W. (2001) Nutritional condition models for elk: which are the most sensitive, accurate, and precise? *Journal of Wildlife Management*, **65**, 988-997.

Coogan, S.C.P, Raubenheimer, D., Stenhouse, G.B. & Nielsen, S.E. (2014) Macronutrient optimization and seasonal diet mixing in a large omniovore, the grizzly bear: a geometric analysis. *PLoS ONE*, **9**, e97968.

Coogan, S.C.P. & Raubenheimer, D. (2016) Might macronutrient requirements influence grizzly bear-human conflict? Insights from nutritional geometry. *Ecosphere*, **7**, e01204.

Dierenfeld, E.S., Alcorn, H.I. & Jacobson, K.L. (2002) Nutrient composition of whole vertebrate prey (excluding fish) fed in zoos. Beltsville (MD): US Department of Agriculture, Agriculture Research Service, National Agriculture Library, Animal Welfare Information Center.

French, S.P., French, M.G. & Knight, R.R. (1994) Grizzly bear use of army cutworm moths in the Yellowstone Ecosystem. *Bears: Their Biology and Management*, **9**, 389-399.

Gerhart, K.L., White, R.G., Cameron, R.D. & Russel, D.E. (1996) Body composition and nutrient reserves of arctic caribou. *Canadian Journal of Zoology*, **74**, 136-146.

Hasan, M.R. & Chakrabarti, R. (2009) Use of algae and aquatic macrophytes as feed in small-scale aquaculture: a review. FAO Fisheries and Aquaculture Technical Paper, No. 531. Food and Agriculture Organization of the United Nations (FAO). Rome, Italy.

Hwangbo, J., Hong, E.C., Jang, A., Kang, H.K., Oh, J.S., Kim, B.W. & B.S. Park. (2009) Utilization of house fly-maggots, a feed supplement in the production of broiler chickens. *Journal of Environmental Biology*, **30**, 609-614.

INRA, CIRAD, AFZ, & FAO. (2017) Feedipedia: animal feed resources information system. Available at: https://www.feedipedia.org/content/about-feedipedia (accessed July 2017)

Kempster, A.J., Cook, G.L. & Grantley-Smith, M. (1986) National estimates of the body composition of British cattle, sheep and pigs with special reference to trends in fatness. A Review. *Meat Science*, **17**, 107-138.

Knudsen, K.E.B. (2001) The nutritional significance of “dietary fibre” analysis. *Animal Feed Science and Technology*, **90**, 3-20.

Pritchard, G.T. & Robbins, C.T. (1990) Digestive and metabolic efficiencies of grizzly and black bears. *Canadian Journal of Zoology*, **68**, 1645-1651.

Robbins, C.T., Moen, A.N. & Reid, J.T. (1974) Body composition of white-tailed deer. Journal of *Animal Science*, **38**, 871-876.

Senior, A. M., Grueber, C. E., Machovsky-Capuska, G., Simpson, S. J. & Raubenheimer, D. (2016) The macronutritional consequences of food generalism in an invasive mammal, the wild boar. *Mammalian Biology*, **81**, 523-526.

Steen, R.W.J., Lavery, N.P., Kilpatrick, D.J. & Porter, M.G. (2003) Effects of pasture and high-concentrate diets on the performance of beef cattle, carcass composition at equal growth rates, and the fatty acid composition of beef. *New Zealand Journal of Agriculture Research*, **46**, 69-81.

Thomas, V.G. & Popko, R. (1981) Fat and protein reserves of wintering and prebreeding rock ptarmigan from south Hudson Bay. *Canadian Journal of Zoology*, **59**, 1205-1211.

US Department of Agriculture, Agriculture Research Service, Nutrient Data Laboratory (2015) USDA National Nutrient Database for Standard Reference, Release 28. Version Current. Available at: https://ndb.nal.usda.gov/ndb/ (accessed July 2017)

Xiaoming, C., Ying, F. Hong, Z., & Zhiyong, C. (2010) Review of the nutritive value of edible insects. In: P.B. Durst, D.V. Johnson, R.N. Leslie, K. Shono, (eds.) Proceeding of a workshop on Asia-Pacific resources and their potential for development. 2008 FEB 19-21; Chiang Mai, Thailand. Bangkok (Thailand): Food and Agriculture Organization of the United Nations Regional Office for Asia and the Pacific. Pp. 85-92.
